# Current status and future perspectives of immunotherapy in Latin America and Cuba

**DOI:** 10.1186/1939-4551-7-28

**Published:** 2014-11-12

**Authors:** Alexander Diaz Rodriguez, Alexis Labrada Rosado, Raúl Lázaro Castro Almarales, Mirta Álvarez Castelló

**Affiliations:** Medical Center: José Manuel Seguí, Artemisa, Cuba; National Center of Bioproducts, BioCen, Mayabeque, Cuba; University Hospital ¨General Calixto García¨, Havana, Cuba

**Keywords:** Immunotherapy, Latin America, Cuba

## Abstract

Most Latin-American countries use subcutaneous immunotherapy (SCIT) extracts from the United States and Europe and sublingual immunotherapy (SLIT) from Europe, with the exception of Argentina, Brazil, Cuba and Mexico. The number of researches on immunotherapy (IT) in Latin America has increased extensively in the last years. Only few Latin American countries have their own guidelines on IT, and, in general, the economic resources for medical research on IT are still low in the area. A global approach for the future of IT in Latin America includes to improve standardization, quality control and the production of allergen products, to develop IT guidelines and clinical investigation by the highest number of countries, to improve the regulatory status for allergens products in the area, and to expand IT accessibility for low-income patients. In Cuba, the first registered allergen vaccines were developed and registered in 2006: a standardized (in biologic units) and freeze dried product for SCIT, with a sublingual version developed in 2009. As much as 23.000 IT treatments were applied in 2011, all provided to patients free of charge. In 2012, Cuban researchers developed an IT vaccine with adjuvant for subcutaneous route, which uses *Neisseria meningitidis* proteoliposome as an adjuvant, added to the purified *Dermatophagoides siboney* major allergens: Der s1 and Der s2. Since December 2012, this vaccine is in Phase I clinical trial, evaluating its safety, tolerability and immunogenicity in asthmatic patients sensitized to this allergen*.* Cuban perspectives on IT includes to work on new indications for IT, to investigate the preventive effect and cost-effectiveness for the current vaccines, to develop new products with mixed formulas of house dust mites for SLIT, to complete the phase I and II clinical study for dust mite plus adjuvant vaccine, to develop allergen vaccines for fungi allergy and to complete the Cuban guideline for allergen IT management.

## Introduction

Allergic diseases represent an economic burden for health system, both in direct and indirect costs. Allergen-specific immunotherapy is one of the most important treatments for allergic disorders, so that the development in this area can be considered a sensitive indicator regarding the advances in the management of allergic diseases in a specific area or country. In addition, allergen-specific immunotherapy is currently recognized as a biological response modifier and the only treatment able to influence the natural course of allergic disease [1].

Several randomized, placebo-controlled studies and meta-analysis have confirmed the effectiveness of immunotherapy in asthma and rhinitis, with a significant reduction in asthma symptoms and medication use, as well as improvement in bronchial hyper-reactivity [2]. Even more, the U.S. Food and Drug Administration (FDA) approves the use of subcutaneous allergen extracts for the treatment of seasonal and perennial allergic rhinitis, allergic asthma, and venom sensitivity and recently approved the use of sublingual allergen extract for the treatment of certain grass pollen allergies [3].

Latin America (LA), the region of the Americas where Romance languages are primarily spoken, includes Mexico, most of Central and South America and in the Caribbean: Cuba, the Dominican Republic, and Puerto Rico. It is compounded by 20 countries and has an area of around 20 Million squared kilometers. Its population is estimated at almost 580 million people, where Brazil, Mexico, Colombia and Argentina are the most populated countries [4, 5].

Cuba, officially the Republic of Cuba, is the main island country in the Caribbean. The island covers an area of approx. 110. 000 square kilometers and has a population of over 11 million people, and the climate is tropical and humid [6].

The purpose of this paper is to describe the present situation and future perspective of immunotherapy in Latin America and Cuba.

## Review

### Latin America

*Dermatophagoides pteronissynus*, *Dermatophagoides farinae*, *Blomia tropicalis*, *Lepidoglyphus* and *Euroglyphus* and are the most prevalent dust mites in LA [7–10]. Pollens are also very common in some areas, with a seasonal pattern in some countries like Argentina and Chile; other important allergens include cockroach and some airborne fungi like Alternaria and Aspergillus [11–16].

Sublingual and subcutaneous IT are practiced in all Latin-American countries, where allergic rhinitis and asthma are their most common indications [17–19]. IT for hymenoptera allergy is also common in the area, but there are few publications about it [20–22]. The majority of Latin-American countries use SCIT extracts from the United States and Europe and 50% of them from local providers. On the other hand, SLIT extracts are mostly from Europe, but some countries like Argentina, Brazil, Cuba and Mexico prepare their own SL vaccines. Last, only a small number of countries fulfill regulatory status for their allergen products in the area; in this case Brazil, Cuba and in some extend, Argentina [17, 23, 24].

The number of studies on immunotherapy (IT) in Latin America has increased extensively in the last few years. The publications on adverse events with IT are beginning to appear. Only few countries have their own guidelines on IT, and, in general, the economic resources for medical research on IT are still low in the area [17].

A global approach for the future of IT in LA could be to improve standardization, quality control and the production of Latin American allergen products, to develop IT guidelines by the highest number of countries and to encourage clinical investigation on IT. Another important points comprises the improvement in regulatory status for allergens products in the area and the expansion in IT accessibility for low-income patients.

### Cuba

There are 281 allergists in Cuba, with a ration of around 40.000 patients per allergist. Most of the Cuban allergists belong to the Cuban Society of Allergy, Asthma and Clinical Immunology, our national affiliation. Currently, we have 99 allergy services, mostly in primary care. There are 11 medicine faculties along the country supporting allergy residency, with 94 allergy professors [25].

It’s been estimated that, at least, the 20% of the Cuban population suffer from allergy. Asthma prevalence is also high, ranging from 9 to 15% in general population. In children, according to the Cuban ISAAC (International Study of Asthma and Allergies in Childhood) Study, the prevalence for asthma, allergic rhinitis and atopic dermatitis in the ages 6-7 and 13-14 years, ranks high in the continent (Table 1) [26].

**Table 1:** **Cuban ISAAC study 2002- 2004** [26]

Disease	6-7 years	13-14 years
Asthma	31.6%	17.6%
Allergic rhinitis	39.8%	38.5%
Atopic dermatitis	22.3%	14%

In Cuba, house dust mites are the most important allergen sources. *Blomia tropicalis, Dermatophagoides pteronyssinus and Dermatophagoides siboney, which is* a variant of *Dermatophagoides farinae* endemic in Cuba, are the most significant species [27–31] (Table 2)*.* Some studies suggest that pollens, cockroaches and fungi could also be important*,* but more evidence is necessary to support it [32–37].

**Table 2:** **Most prevalent allergens in Cuba** [27–31]

Most prevalent allergens in Cuba	
**Dust mites**	*Dermatophagoides pteronyssinus*
	*Dermatophagoides siboney* (endemic)
	*Blomia tropicalis*
**Pollens**	*Cynodon dactylon* (Bermuda grass)
	*Parthenium hysterophorus* (escoba amarga)
	*Bidens pilosa* (yellow grass)
**Molds**	*Penicillium notatum*
	*Cladosporium herbarum*
	*Alternaria tenuis*
**Insects**	Cockroaches (*Periplaneta americana*)

In the nineties, a biotech pole was created in Havana, and then in other provinces, including important scientific institutions and industries. All these centers work together on the application of Biotechnology into healthcare, developing and manufacturing vaccines and other biologic products. Cuban biotech pole involve more than 30 institutions, 15.000 workers, nearly 2.000 researchers and more than 150 research projects. There is a group of leading centers among them: the Center of Genetic Engineering and Biotechnology, the Center of Molecular Immunology and the National Center of Bioproducts (BioCen) [38]. The latter, also called BIOCEN is the place where the allergen-specific vaccines are produced at national scale.

To date, three standardized allergenic extracts (*Blomia tropicalis, Dermatophagoides pteronyssinus and Dermatophagoides siboney)* have been licensed in Cuba for their use as *in-vivo* diagnosis and specific immunotherapy for allergic diseases. These allergen vaccines were developed and registered in 2006: a standardized (in biologic units), freeze dried product for SCIT and the sublingual version was developed in 2009. In 2008, the vaccines were included into the group of basic medications by the Cuban Ministry of Health and the SL variant was incorporated in 2009. In 2011, as much as 23.000 IT treatments were provided to patients free of charge. About half the total IT vaccines were prescribed by sublingual route. The potency of Biocen vaccines were compared with Diater and ALK products with no statistically significant differences between them [39].

**Figure 1:**
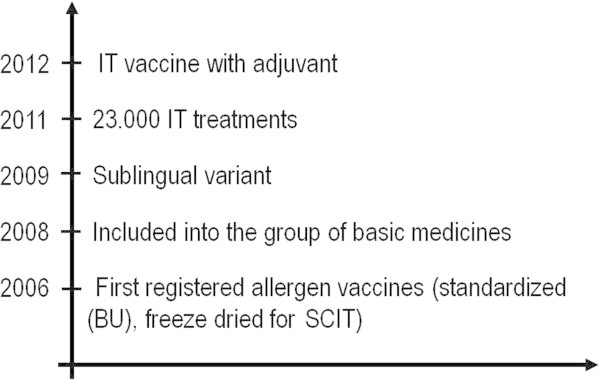
**Chronology of development of immunotherapy in Cuba.**

In 2012, a group of Cuban researchers developed an IT vaccine with adjuvant for subcutaneous route (Figure 1) [40–44]. This new vaccine uses *Neisseria meningitidis* proteoliposome, a bacterial product, as an adjuvant, added to the purified *Dermatophagoides siboney* major allergens: Der s1 and Der s2. From December 2012, this vaccine is in Phase I clinical trial to evaluate safety, tolerability and immunogenicity in asthmatic patients sensitized to this allergen. Another vaccine with the same adjuvant is also being developed with *Blomia tropicalis.*

There are 11 randomized, controlled clinical trials on IT (9 already finished), all registered at the Cuban public registry for clinical trials [45], available at URL: http://rpcec.sld.cu/en/home. The first clinical trial applied in children is still in progress: a dose –response study by SL route. All these trials have used mite vaccines, and have had asthma as a target. There are also 3 studies on IT adverse events. Cuban allergists are currently working on their own guideline for allergen IT management. Also related, the Cuban Society of Allergology endorsed and co-organized, with the Cuban Immunology Society, two international meetings on allergen vaccines in 2009 and 2012.

Subcutaneous immunotherapy with *Blomia tropicalis, Dermatophagoides pteronyssinus and Dermatophagoides siboney,* the most prevalent Cuban dust mites, has proved to be effective and safe for the control and amelioration of asthma symptoms in asthmatic patients [40, 42]. The therapeutic effect and safety of *Dermatophagoides pteronyssinus*, *Dermatophagoides siboney* and *Blomia tropicalis* was also demonstrated in asthmatic patients using SL route. Both clinical symptoms and medication intake were reduced compared to placebo. Allergen skin sensitivity also decreased significantly (p < 0.01). PEF (peak expiratory flow) variability also diminished significantly (p < 0.05). The treatment was considered effective in 77% of patients. Local reactions were noted only in 0.43% of administrations and no systemic reactions were observed [41].

The Cuban perspectives on IT include to work on new indications for IT, to investigate the preventive effect and cost-effectiveness for current vaccines, to develop new products with mixed formulas of house dust mites for SLIT, to complete the phase I and II clinical study for dust mite plus adjuvant vaccine, to develop allergen vaccines for fungi allergy in asthmatics and to complete the Cuban guideline for allergen IT management.

## Conclusion

Although most Latin American countries and Cuba are developing countries, much has been achieved in terms of treatment and research in immunotherapy, however, there are still some points requiring improvement in regard to immunotherapy in the area.

## Abbreviations

LA: Latin America
SC: Subcutaneous
SL: Sublingual
IT: Immunotherapy
Der s1: *Dermatophagoides siboney* major allergen 1
Der s2: *Dermatophagoides siboney* major allergen 2
ISAAC: International study of asthma and allergies in childhood
BioCen: National center of bioproducts
PEF: Peak expiratory flow.
